# Roles for growth factors and mutations in metastatic dissemination

**DOI:** 10.1042/BST20210048

**Published:** 2021-06-08

**Authors:** Nishanth Belugali Nataraj, Ilaria Marrocco, Yosef Yarden

**Affiliations:** Department of Biological Regulation, Weizmann Institute of Science, Rehovot, Israel

**Keywords:** epithelial-to-mesenchymal transition, growth factors, metastasis, mutation, tumor microenvironments, wound healing

## Abstract

Cancer is initiated largely by specific cohorts of genetic aberrations, which are generated by mutagens and often mimic active growth factor receptors, or downstream effectors. Once initiated cells outgrow and attract blood vessels, a multi-step process, called metastasis, disseminates cancer cells primarily through vascular routes. The major steps of the metastatic cascade comprise intravasation into blood vessels, circulation as single or collectives of cells, and eventual colonization of distant organs. Herein, we consider metastasis as a multi-step process that seized principles and molecular players employed by physiological processes, such as tissue regeneration and migration of neural crest progenitors. Our discussion contrasts the irreversible nature of mutagenesis, which establishes primary tumors, and the reversible epigenetic processes (e.g. epithelial–mesenchymal transition) underlying the establishment of micro-metastases and secondary tumors. Interestingly, analyses of sequencing data from untreated metastases inferred depletion of putative driver mutations among metastases, in line with the pivotal role played by growth factors and epigenetic processes in metastasis. Conceivably, driver mutations may not confer the same advantage in the microenvironment of the primary tumor and of the colonization site, hence phenotypic plasticity rather than rigid cellular states hardwired by mutations becomes advantageous during metastasis. We review the latest reported examples of growth factors harnessed by the metastatic cascade, with the goal of identifying opportunities for anti-metastasis interventions. In summary, because the overwhelming majority of cancer-associated deaths are caused by metastatic disease, understanding the complexity of metastasis, especially the roles played by growth factors, is vital for preventing, diagnosing and treating metastasis.

## Introduction

**Figure 1.**
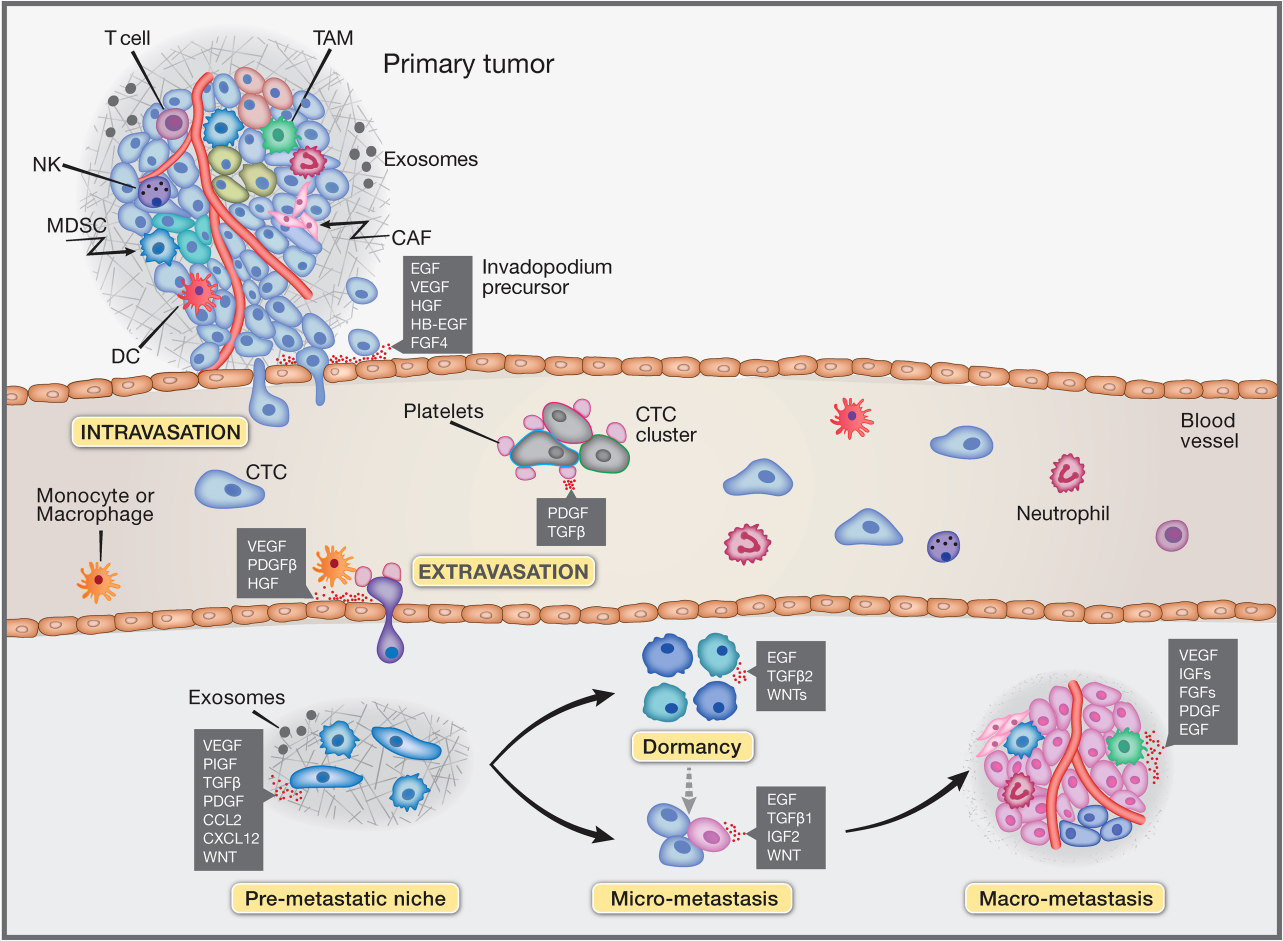
Schematic model of the metastatic spread of cancer cells. Primary tumor cells are embedded in a microenvironment comprising stromal cells, such as cancer-associated fibroblasts (CAFs), myeloid-derived suppressor cells (MDSC), dendritic cells (DCs), natural killer (NK) cells, T cells, tumor-associated macrophages (TAMs), neutrophils, along with blood vessels. Shedding of tumor cells and their invasion across tissue barriers permit establishment of circulating tumor cells (CTCs) and complete the process called intravasation. While in blood vessels, CTC clusters and solitary cells are protected by platelets and neutrophils, which facilitate extravasation. Tumor-secreted soluble factors, tumor-shed extracellular vesicles (exosomes) and bone marrow-derived cells (BMDCs) co-operate to form the pre-metastatic niche. Micro-metastases often experience a variable length dormancy phase, which might lead to cancer cell death. An angiogenic switch can initiate outgrowth and formation of secondary tumors. Growth factors (shown as red dots) and cytokines involved in each step of the cascade are indicated in the corresponding boxes. The abbreviations used are: CCL2, C-C motif chemokine ligand 2; CXCL12, C-X-C motif chemokine ligand 12; EGF, epidermal growth factor; FGF, fibroblast growth factor; HB-EGF, heparin-binding EGF-like growth factor; HGF, hepatocyte growth factor; IGF, insulin like growth factor; PDGF, platelet-derived growth factor; PIGF, placental growth factor; TGFβ, transforming growth factor beta; VEGF, vascular endothelial growth factor; WNTs, Wnt family members.

The overwhelming majority of cancer-associated deaths (>90%) are caused by metastatic disease, rather than the respective primary tumors [1,2]. Hence, understanding the complexity of this process is vital for optimizing the treatment of patients with advanced malignancies. Already in 1889, Stephen Paget proposed that metastasis depends on cross-talk between cancer cells (the ‘seeds’) and specific organ microenvironments (the ‘soil’). However, despite intensive and prolonged research, the multi-step process of metastasis (often called the ‘invasion-metastasis cascade’ [3]) (Figure 1) remains poorly understood. This is in sharp contrast with the preceding steps of cancer progression, such as tumor initiation and primary tumor growth. Stated differently, while it is clear that somatically acquired ‘driver’ mutations, which affect oncogenes or tumor suppressor genes, propel the initial steps of malignancy, and their stepwise accumulation dictates the pace of tumor progression [4], the effects of genome aberration on metastasis appear less prominent. Instead, studies performed in the last two decades have highlighted control by epigenetic and other reversible processes, such as epithelial–mesenchymal transition (EMT), chemotaxis and a plethora of reciprocal (paracrine) cell-to-cell interactions regulated by soluble factors [5]. Herein, we consider the metastasis cascade as a multi-step process that seized the major principles and main molecular players employed by physiological rather than pathological processes. Such processes include reactions underlying tissue regeneration in wound healing and migration of neural crest (NC) progenitors in development. Our discussion is most relevant to the metastasis of carcinomas, which account for the majority of cancer mortality. We conclude by contrasting the irreversible nature of mutagenesis, which establishes primary tumors, and the phenotypically plastic and reversible processes underlying the establishment of micro-metastases and secondary tumors.

## Principles borrowed from tissue regeneration

The major steps of the metastasis cascade comprise dissemination of cancer cells from the tumor of origin, subsequent intravasation into blood and lymph vessels, and eventual colonization of distant organs. Thus, the opening steps require breaching the basement membrane, which underlies large clusters of initiated cancer cells harboring pioneer mutations. Prior to this step, the primary tumor is considered a non-malignant lesion. For example, ductal carcinoma *in situ* (DCIS) is non-invasive breast cancer that encompasses a wide spectrum of diseases [6]. DCIS is characterized histologically by the rapid proliferation of initiated epithelial cells that are still bounded by the basement membrane of the breast ducts. Once epithelial integrity breaks and the underlying basement membrane is damaged, the surrounding tissue responds by activating a regenerative cascade analogous to the reaction of the skin to mechanical damage. A well-characterized marker of damaged epithelial progenitors is L1CAM, which is required for regeneration of the epithelium and whose abundance in primary tumors is associated with poor disease outcome [7]. Natural wound healing proceeds through several phases involving an inflammatory response and associated cellular migration, proliferation, matrix deposition and tissue remodeling [8]. Activin and other members of the transforming growth factor beta (TGF-b) family play pivotal roles in both wound healing and cancer progression [9]. These processes are enabled, in part, by the ability of activin to control fibroblast proliferation and migration, as well as regulate the production of extracellular matrix (ECM) proteins and collagen cross-linking. In addition to fibroblasts and keratinocytes, macrophages, neutrophils and platelets are attracted to the wound and secrete EGF, VEGF, IL-1 and TGF-b, among other cytokines. In a similar way, the reactive stroma of invasive carcinomas releases various signals and displays markers of immune responses, as well as hypoxic responses [10]. As an outcome of hypoxia, blood vessels are attracted to invasive clusters of cells, such that angiogenesis is a major hallmark shared by both advanced tumors and deep wounds.

## Subversion of progenitor migration during embryogenesis

Although hematogenous dissemination of cancer cells migrating within vascular channels is the best-studied route of metastatic dissemination, alternative pathways of distant colonization exist, but their research is hampered by technical issues. For example, cancer cells can migrate along nerves in a process called perineural invasion, which requires neoplastic invasion of nerves. The presence of cancer cells in the perineurium has been associated with poor prognosis, metastasis to lymph nodes and high recurrence of colorectal and other tumors [11,12]. Similarly, the abluminal surface of lymphatic and blood vessels (angiotropism) enables the spread of melanoma via extravascular migratory routes, while avoiding intravasation into vascular routes [13]. A recent report made use of an oncogenic BRAF mutation in mice with transgenic HGF overexpression and an oncogenic CDK4 germline mutation [14]. Interestingly, the authors reported that HGF–MET signaling and oncogenic BRAF can collaborate by enhancing angiotropic growth at the invasive front of primary tumors and in metastatic lesions of the lung. Notably, both perineural invasion and the extravascular migratory route avoid the shear force experienced by tumor cells, which is characteristic to hematogenous dissemination.

Unlike hematogenous dissemination, extravascular migration of embryonic progenitors widely occurs in morphogenesis. Perhaps the best understood and the one most relevant to metastasis is the migration of NC cells in the developing embryo [15]. The NC is a highly migratory embryonic cell population that develops into numerous cell lineages, including melanocytes, smooth muscles, neurons and the craniofacial mesenchyme. Importantly, NC cells migrate along well-defined routes in the developing embryo and display an enormous ability to invade tissues and organs. After the NC forms along the border of the neural plate, NC cells undergo EMT, whereby polarized epithelial cells lose adhesion and adopt mesenchymal morphologies in preparation for migration [16]. Many of the processes underlying the formation and migration of NC cells can also play critical roles in cancer progression [17]. For example, both NC cells and circulating tumor cells (CTCs) form loosely associated collectives and use both guidance cues and the local ECM to collectively migrate. In breast cancer, collective invasion represents the predominant invasion mode and associates with distant metastasis [18]. The use of mouse models with tagged mammary tumors provided important insights: although rare in the circulation compared with single CTCs, CTC clusters have up to 50-fold increased metastatic potential [19]. These studies also identified the cell junction component plakoglobin as highly differentially expressed, such that knockdown of plakoglobin abrogated CTC cluster formation and suppressed lung metastases.

## Cell-to-cell interactions while CTCs are traveling in the circulation

While in transit, CTCs are vulnerable to immune attacks, hydrodynamic flow and shear stress, but interactions with specific cells, especially platelets, neutrophils, monocytes and endothelial cells, might support their survival and facilitate eventual extravasation [1]. First noted by the French physician Armand Trousseau, increased incidence of venous thrombosis associated with certain cancers (Trousseau's syndrome) [20]. The molecular basis of Trousseau's syndrome has been attributed to the ability of platelets to protect tumor cells in circulation from the normal immune response or natural killer cells [21]. In addition, platelet-derived lysophosphatidic acid (LPA) can support and activate metastatic breast cancer cells, as well as stimulate the release from cancer cells of interleukin-6 and interleukin-8, leading to bone destruction and further supporting metastatic growth [22]. Of note, LPA is a natural bioactive lipid with growth factor-like functions due to activation of a series of six G protein-coupled receptors (LPA_1–6_). LPA receptor type 1 (LPA_1_) signaling has been associated with the up-regulation of heparin-binding EGF-like growth factor (HB-EGF) [23]. Accordingly, analysis of primary tumors of a large cohort of breast cancer patients found a significantly higher expression of HB-EGF in breast tumors expressing high levels of LPA_1_. These results raise the possibility of using anti-HB-EGF antibodies or LPA_1_ antagonists to inhibit metastasis.

Although neutrophils — the most abundant leukocytes in human blood — can kill disseminated cancer cells under certain conditions, CTCs can recruit neutrophils to promote metastasis. For instance, neutrophils can enhance the extravasation of tumor cells, mainly through the secretion of matrix metalloproteinases [24]. One way enabling neutrophils to kill harmful microorganisms entails the formation of neutrophil extracellular traps (NETs), which are DNA meshes released into the extracellular space, where they trap microorganisms. NETs form in human pancreatic, liver and gastric cancer, as well as within the vasculature. In animal models, NET-like structures form around metastatic cells and stimulate the invasion and migration of breast cancer cells, implying that induction of NETs by cancer cells promotes metastasis [25]. Importantly, G-CSF, a key regulator of neutrophil production and a potent mobilizer of haemopoietic stem cells, emerged as a critical factor in the induction of NETs by cancer cells.

## Cancer cell extravasation

Cancer cell extravasation usually occurs in small capillaries, where CTCs are physically trapped by size restriction. Once pressed into small capillaries, CTCs are deformed and undergo mechanical stress that is responsible for the loss of up to 90% of cancer cells entering small vessels [26]. Nevertheless, subsets of cancer cells eventually infiltrate the parenchyma of organs to seed metastatic colonies. The underlying process, termed transendothelial migration (TEM), depends on disruptors of vascular integrity, such as VEGF, metalloproteinases and many pairs of ligand–receptor molecules, such as selectins, integrins, cadherins and CD44. In addition, a distinct population of host macrophages is recruited to metastatic cells and enables efficient tumor cell extravasation [27]. Several studies revealed that local death of endothelial cells can enhance extravasation, for example by means of ATP released by dying cells. It has been shown that the release of ATP can enhance the survival of tumor cells in the face of mechanical stress [28]. Interestingly, human and murine tumor cells induce programmed necrosis (necroptosis) of endothelial cells, which promotes tumor cell extravasation and metastasis [29]. Unexpectedly, tumor-cell-induced endothelial necroptosis requires amyloid precursor protein expressed by tumor cells and its receptor, death receptor 6 (DR6), on endothelial cells, as the primary mediators of necroptotic signaling.

## Organ-specific metastatic potential and the pre-metastatic niche

It is widely accepted that metastasis is an inefficient process whereby the vast majority of CTCs are not able to survive and proliferate at distant sites. In addition, metastatic cells often display tissue and organ tropism, in a way that cannot be explained by circulatory patterns. Whether or not organ-specific metastasis potential is driven by the accumulation of genetic mutations or by epigenetic events, such as up-regulation of adhesion molecules or other factors, remains unclear [3]. For example, breast cancer metastases frequently colonize the bone and the lung, and less frequently the liver and brain. By means of sequential isolation of isogenic mammary tumor cells that preferentially infiltrate the brain, it became clear that cell-surface glycosylation dictates organ-specific metastatic interactions [30]. Along with other genes, *HB-EGF* emerged from this study as an enhancer of brain metastasis, as opposed to metastasis to liver or lymph nodes. Interestingly, another EGFR ligand, epiregulin, was found to be included in an 18-gene lung metastasis signature [31]. According to an alternative view, a favorable microenvironment, called the pre-metastatic niche (PMN), located in an organ distant from a primary tumor, is critical for tumor metastasis and target tissue selection. PMN establishment requires both extracellular vesicles (exosomes) [32] and bone marrow-derived haematopoietic progenitor cells. The latter cells express vascular endothelial growth factor receptor 1 (VEGFR1/Flt1) and form cellular clusters before the arrival of tumor cells [33]. In addition to bone marrow-derived cells, cancer-derived factors, and ECM, a host of growth factors and pro-inflammatory cytokines are involved [34]. The list includes VEGF, WNT ligands, interleukin (IL)-6, IL-1β, CC-chemokine ligand 2 (CCL2), granulocyte-colony-stimulating factor (G-CSF), granulocyte-macrophage colony-stimulating factor (GM-CSF), stromal cell-derived factor (SDF)-1, macrophage migration inhibitory factor (MIF) and Chemokine (C-X-C motif) ligand 1 (CXCL1; see boxes in Figure 1).

## Metastasis-driving genetic aberrations versus phenotypic plasticity

In accord with the large contribution of metastasis to the incidence of oncology-related deaths, it has been reported that predictors of cellular migration, rather than proliferation of breast cancer cells, are strongly associated with patient survival [35]. Surprisingly, however, only very few driver mutations have been directly linked to the metastatic cascade. For example, a recent screen aimed at putative driver mutations escalating the risk of metastasis identified three mutations in nucleoporin 93 (NUP93), a component of nuclear core complexes [36], which is involved in 3D migration and reorganization of the actin cytoskeleton [37]. Guided by the principle that cancer, including metastasis, involves the evolution of malignant cells attempting to survive under the stresses of tumor progression [38], larger-scale surveys of cancer genomics have tried identifying pro-metastatic mutations [2]. Apparently, there are two general scenarios of metastatic dissemination, and both have been supported by genome-wide analyses: In the linear progression model, the metastasis-competent clone arises late in tumorigenesis and disseminates just before clinical detection of the primary lesion. Hence, the expected genetic divergence between the primary tumor and its metastasis (termed P-M genetic divergence) might be rather limited [38]. In the parallel progression model, the metastatic clone disseminates from the primary tumor early on, and both the primary tumor and the metastases continue to evolve in parallel. As a result, the P-M genetic divergence might be substantial. Sampling and exposure to systemic therapy might confound the inference of the exact mode of metastatic spread, especially because therapy frequently associates with adaptive mutagenesis [39]. To circumvent this, Reiter and colleagues analyzed sequencing data from untreated metastases and inferred cancer phylogenies [40]. Their analyses unveiled depletion of putative driver mutations among metastases, and the majority of those that were observed had only weak or no predicted functional effects. These observations are consistent with the ‘Big Bang’ model proposed by Christina Curtis and colleagues [41]. Accordingly, tumors grow predominantly as a single expansion producing many subclones that are not subject to stringent selection, and where both clonal and subclonal alterations arise early during growth.

## Roles for EMT, MET and other epigenetic switches in metastasis

Several reasons might explain why inter-metastatic heterogeneity is relatively low, while paracrine loops involving growth factors are widespread. For one, under certain conditions, disseminated cancer cells enter proliferative quiescence, such that the tumor becomes dormant (e.g. due to failure to activate angiogenesis). In addition, driver gene mutations may not confer the same advantage in the microenvironment of the primary tumor and of a distant site. Stated differently, phenotypic plasticity and epigenetic events, rather than rigid cellular states hardwired by mutations, might become advantageous at the late stages of tumor progression. The metastasis cascade provides several examples of reversible changes. While the invasion and dissemination steps during carcinoma progression have been associated with EMT, the reverse process — mesenchymal-epithelial transition (MET) — likely propels the outgrowth of cancer cells once they settled at distant sites. For example, repression of the EMT inducer Prrx1 is essential for metastatic colonization [42]. In similarity, activation of another EMT-inducing transcription factor, Twist1, is sufficient for stimulating carcinoma cells to undergo EMT. However, reversion of this process is essential for the proliferation of disseminating tumor cells at distant organs [43]. Notably, whether or not EMT is obligatory for metastasis remains a longstanding source of debate. For instance, inhibiting EMT by overexpressing the microRNA 200 did not affect spontaneous breast-to-lung metastasis [44], and mouse models of pancreatic ductal adenocarcinoma (PDAC) with deletion of Snail or Twist did not alter systemic dissemination or metastasis [45]. This might be explained by the transient nature of EMT or by the existence of a novel metastasis program, a possibility raised by experiments that made use of mesenchymal cell reporter mice and a PDAC model [46].

Viewed from a slightly different angle, emerging data show that EMT comprises a spectrum of intermediate states, coined as hybrid or partial EMT. Hybrid EMT cells exhibit high tumorigenic properties, leading to stemness and therapy resistance [47]. Therefore, it has been argued that these cells are more prone to colonize distant organs. Using a lineage-labeled mouse model of PDAC, Aiello et al. [48] studied EMT *in vivo* and reported that most tumors lose their epithelial phenotype to acquire the ‘partial EMT’ phenotype . Importantly, carcinoma cells utilizing this program migrate as clusters, which contrast with the single-cell migration pattern associated with EMT mechanisms. According to another report, adherens junctions and the EMT–MET interconversion play roles in metastatic organotropism of pancreatic cancer [49]. Furthermore, it has been observed that loss of FAT1, which encodes a protocadherin, increased tumor stemness and spontaneous metastasis, through the induction of a hybrid EMT state [50].

In conclusion, reversible EMT appears essential for tumor metastasis. Cellular plasticity extends to tumor-initiating stem cells and the ability to form metastases. For instance, hyperactivation of the WNT signaling pathway turns intestinal LGR5^+^ cells into tumor-initiating cancer stem cells (CSCs), but the cells that disseminate from the primary tumor and seed liver metastasis in mouse models are predominantly LGR5^–^ [51]. Still, once established in the liver, some proliferating metastatic cells re-acquire LGR5 expression. Apart from WNT, this pathway might be activated by EGF family growth factors [52] and other growth factors capable of stimulating SMAD3 [53], which demonstrates the versatility and plasticity of LGR5 induction and the metastatic cascade. To exemplify the scope and variety of the contribution of epigenetic switches and soluble factors to metastasis, below we describe several recent examples and provide a longer list of growth factors in Table 1.

**Table 1.** Roles for the major growth factors in metastasis

GF family	Receptors	Roles in metastasis
**EGF and NRG** EGF TGFα NRG 1-4 AREG EREG HB-EGF EPIGEN	**EGFR, HER2, HER3, HER4**	**Constitutive activation and overexpression of ERBB/HER receptors are correlated with poor prognosis, drug resistance, cancer metastasis, and shorter patient survival rates in several types of cancer [69,70].** • EGF secreted by M2-like TAMs suppresses lncRNA LIMT expression via activating EGFR-ERK signaling pathway to promote ovarian cancer metastasis [71]. • HER2 and EGFR promote prostate cancer metastasis to bone [72]. • TFCP2 induces TNBC progression via a positive feedback loop comprising EGF/TGFα and the AKT signaling axis [73]. • A TNFα-TGFα-EGFR interacting loop between tumor and stromal cells promotes peritoneal metastasis of ovarian cancer [74]. • Cross-talk between the Hippo–YAP pathway and the heterodimer kinase complex formed by ROR1 with HER3 promotes breast cancer bone metastasis in a lncRNA-dependent manner [75]. • NRG1-HER4-YAP signaling contributes to migration of breast cancer cells [76]. • NRG1 in luminal breast cancer defines pro-fibrotic and migratory CAFs [77]. • Tumor microenvironment-derived NRG1 promotes antiandrogen resistance in prostate cancer [78]. • AREG/EGFR signaling enhances the suppressive function of Treg cells and promotes tumor metastasis [79]. • AREG overexpression induces a phenotypic switch from malignant ascites to solid metastatic phenotype in a cell clone obtained from a patient derived-ovarian cancer model [80]. • Autocrine epiregulin contributes to lung metastasis via EMT in salivary adenoid cystic carcinoma through activation of EGFR [81]. • EREG is essential for transformation of fibroblasts to CAFs, which is required to induce EMT and invasion via the JAK2-STAT3 pathway in OSCC [82]. • Inhibition of the HB-EGF/EGFR pathway using peptides inhibited HB-EGF-induced ovarian cancer cell migration and invasion [83]. • Tumor cell clusters produce epigen, which controls switching between collective migratory and proliferative modes. Epigen knockdown strongly reduces metastatic outgrowth [60].
**TGF-β** TGF-β1-3 BMPs	**TGF-βR1,2**	**TGF-β plays a dual role during tumorigenesis; in early stages of carcinogenesis, it induces growth inhibition, but in the late stages it promotes cancer progression and metastasis, due to its ability to dedifferentiate many cell types, suppress immune cell development and indirectly allow vascular growth [84,85].** • TGF-β and MMPs produced by myeloid cells in advanced-stage cancer inhibit antitumor immune reactions and promote metastasis [86]. • By repressing the expression of miR-211, adipocytes sensitize melanoma cells to TGF-β signaling, leading to a switch from a proliferative to an invasive phenotype [87]. • EGFR, AP-1, p63, and TGF-β co-operate to promote invasiveness of breast cancers [88]. • TGF-β-induced DACT1 biomolecular condensates repress WNT signaling to promote bone metastasis [89]. • Activation of the canonical BMP4-SMAD7 signaling pathway blocks breast cancer metastasis [90].
**VEGF** VEGF-A VEGF-B VEGF-C VEGF-D	**VEGFR1-3**	**VEGFs plays a role in tumor-associated angiogenesis, tissue infiltration, and metastasis formation [91,92].** • VEGF-A secreted by primary tumors causes vascular hyperpermeability in pre-metastatic lung via the occludin phosphorylation/ubiquitination pathway [93]. • Suspending anti-VEGF therapy induces metastasis through a liver revascularization mechanism [94]. • VEGF-A expression promotes fibrosarcoma metastasis [95]. • VEGF-B promotes cancer metastasis through the remodeling of tumor microvasculature [96]. • VEGF-C and FGF-2, collaboratively promote angiogenesis, lymphangiogenesis and lymph-node metastases [97]. • By inducing EMT, epithelial breast cancer cell cross-talk, VEGF-C promotes tumor growth and metastasis [98]. • VEGF-C up-regulation induced by c-MYC promotes lymphatic metastasis of pancreatic neuroendocrine tumors [99]. • Breast cancer cell subclones expressing IL-11 and FIGF (VEGF-D) promote formation of polyclonal metastases composed of driver and neutral subclones [100].
**IGF** IGF1 IGF2	**IGF1R, IGF2R**	**IGF signaling promotes cancer progression by affecting tumorigenesis, metastasis and resistance to cancer therapies [101,102].** • CAF-derived IGF-1 primes breast cancer cells for bone metastasis [103]. • CAFs induce invasion of PDAC cells through paracrine IGF1/IGF1R signaling [104]. • NDRG1 inhibits PSC-CM-induced migration of PaCa via inhibition of HGF/c-MET, IGF-1/IGF-1R signaling [105]. • IGF2 secreted by pericyte promotes formation of breast cancer brain metastasis [65].
**HGF** HGF	**MET, RON**	**HGF/MET signaling promotes EMT and cancer invasiveness [106]**. • Inhibition of HGF and MET together with chemotherapy prevented metastasis in a model of PaCa [107]. • HGF/MET controls EMT and metastasis via FOSL2 in NSCLC [108]. • HGF-mediated cross-talk between CAFs and gastric cancer cells activates metastasis [109]. • Breast cancer cells with high metastatic potential are hypersensitive to HGF secreted by macrophages [110]. • MET signaling activates an inflammatory microenvironment in the brain and facilitates breast cancer metastasis to the brain [111].
**FGF** FGF 1-23	**FGFR1-4**	**FGFs are considered cancer drivers for their ability to regulate angiogenesis, cell proliferation and metastasis [112,113].** • Loss of TGF-β signaling in osteoblasts increases basic FGF to promote bone metastases in an animal prostate cancer model [114]. • Activation of FGF2/FGFR1 promotes cell proliferation, EMT and metastasis in FGFR1-amplified lung cancer via the FGFR1-ERK1/2-SOX2 axis [115]. • Collective migration of parapineal cells is mediated by FGF signaling [116].
**PDGF** PDGF-AA PDGF-BB PDGF-CC PDGF-DD PDGF-A/B	**PDGFRα,β**	**By regulating mesenchymal cells such as fibroblasts, pericytes and smooth muscle cells, PDGFs and their receptors can promote tumor metastasis [117,118].** • Loss of miRNA let-7d and gain of HIF1 activity promote breast cancer brain metastasis via PDGF; inhibition of PDGFR suppresses brain metastasis [119]. • In pericytes and stromal fibroblasts, the PDGF-BB-PDGFRβ-IL-33-ST2 axis recruits TAMs and induces metastasis [120]. • PDGF-C secreted by CAFs promotes GIST growth and metastasis [121]. • PDGF-BB induces pericyte-fibroblast transition (PFT), contributing to tumor invasion and metastasis [122].
**CSF1 (M-CSF)**	**CSF1R**	**CSF1/CSF1R signaling indirectly promotes tumor progression by regulating the functions of macrophages, or directly, by promoting release of inflammatory mediators from tumor cells [123].** • Co-overexpression of TWIST1 and CSF1 promotes OSCC invasiveness [124]. • The Oct4/CSF1 axis promotes M2 macrophage polarization, leading to lung cancer growth and metastasis [125]. • miR-149 acts as a metastasis-suppressing microRNA in breast cancer cells by reducing recruitment and M2-polarization of macrophages induced by CSF1 [126].
**WNT**	**FZDs** **Co-receptors:** **LRP 5/6** **Ryk** **ROR 1/2**	**Both canonical and non-canonical WNT pathways in the tumor microenvironment contribute to EMT, metastasis and cancer stem cell maintenance in lung, colorectal and breast cancers [127].** • The WNT pathway acts as a driver of bone metastatic invasion of prostate cancer [128]. • The canonical WNT/β-catenin/Slug pathway contributes to cancer cell invasion and lymph-node metastasis of HNSCC [129]. • Autocrine WNT7b plays a role in CRC metastasis by promoting EMT through the WNT/β-catenin signaling pathway [130].
**CXCL12**	**CXCR4**	**The CXCL12/CXCR4 axis regulates metastasis via different mechanisms (e.g. EMT and up-regulation of metalloproteinases) in several cancers, including pancreatic, colon and melanoma [131,132].** • The CXCR4-LASP1 axis enhances the stability of nuclear Snail1, to promote invasion of TNBC cells [133]. • CXCL12/CXCR4 promotes invasion of ovarian cancer cells by means of suppressing ARHGAP10 expression [134]. • CXCR4 signaling promotes the interaction between tumor cells and neutrophils, to regulate onset of metastasis [135]. • Inhibition of DPP-4 facilitates breast cancer metastasis by activating the CXCL12/CXCR4/mTOR axis and promoting EMT [136]. • Cross-talk between fibroblasts derived CXCL12 and endothelial cells promotes tumor cell intravasation, leading to metastasis [137]. • PDGFRα induces SCC metastasis to the lungs through induction and secretion of SDF-1 (CXCL12), with consequent activation of CXCL12/CXCR4 signaling [138].
**NGF**	**TrkA** **P75NTR**	• NGF involved in the mediation of brain metastases and enhanced survival [139].
**HDGF**		• The HDGF-ALCAM axis promotes metastasis of Ewing sarcoma by means of regulating GTPases [140].

Listed are the major classes of growth factors engaged in metastasis, their high-affinity receptors (primarily receptors harboring tyrosine kinase functions), and recently reported functions. The abbreviations used are listed below: CAFs, cancer-associated fibroblasts; EMT, epithelial–mesenchymal transition; GIST, gastrointestinal stromal tumors; HDGF, hepatoma-derived growth factor; HNSCC, head and neck squamous cell carcinoma; NGF, nerve growth factor; NSCLC, non-small cell lung cancer; PaCa, pancreatic cancer; PDAC, pancreatic ductal adenocarcinoma; PSC-CM, pancreatic stellate cell-conditioned media; ROR1, receptor tyrosine kinase-like orphan receptor-1; SCC, squamous cell carcinoma; TAMs, tumor-associated macrophages; TNBC, triple-negative breast cancer.

## Diverse mechanisms enable regulation of metastasis by growth factors

### Growth factors regulate the biochemistry and mechanics of metastasis

Several actin-filled protrusions facilitate migration and invasion of cancer cells. They include lamellipodia and filopodia at the leading edge, and invadopodia facilitating invasion through the epithelium and basal membrane [54]. Invasion across tissue barriers requires cell softening, which is, surprisingly, preceded by transient accumulation of actin stress fibers and cell stiffening [55]. Invadopodia formation is regulated by growth factors and signals from the ECM [56,57]. Growth factor- and oncogene-activated cells are characterized by active PI3K (phosphoinositol 3-kinase) and elevated levels of the phosphoinositol lipid PI(3,4,5)P_3_. Dephosphorylation of this lipid by 5′-phosphatases, such as synaptojanin 2 (SYNJ2), generates PI(3,4)P_2_, which recruits to the plasma membrane an adaptor, TKS5, necessary for the nucleation of invadopodia. SYNJ2 is transcriptionally up-regulated on the treatment of mammary cells with EGF. In addition, SYNJ2 is encoded at 6q25, a chromosomal locus amplified in aggressive forms of breast cancer [58]. In line with this, several reports linked SYNJ2 to invasiveness of glioma and other cancer cells. Normally, two tumor suppressor phosphatases, PTEN and INPP4B, deplete PI(3,4)P_2_ [59] and balance the oncogenic alliance formed by PI3K and SYNJ2. In summary, by regulating inositol lipids and the actin cytoskeleton, growth factors can enhance invasiveness during both intravasation and extravasation.

### Clustering of CTCs is enhanced by growth factors

Clustering of CTCs confers an advantage in terms of successful colonization of distant organs, but the underlying mechanisms are incompletely understood. CTC aggregation depends on cell-to-cell adhesion molecules, such as plakoglobin, CD44 and E-cadherin [19]. According to a recent report, CTCs switch between proliferative and migratory states based on the concentration of a growth factor, epigen, found in nanoscale intercellular compartments present within the clusters [60]. Initially, when applying RNA-seq on tumor cells of different aggregation conditions, the authors found that epigen (along with amphiregulin) was the most induced gene upon tumor cell clustering. Furthermore, depletion of epigen using RNA interference indicated that this growth factor is not required for cell clustering but it might support metastatic outgrowth. Further analyses showed that epigen, a low-affinity ligand of EGFR, was highly concentrated in intercellular sealed cavities. Interestingly, similar principles might permit another family of growth factors to control morphogenesis during embryonic development: Durdu et al. [61] provided evidence for a model in which diffusible signals in the form of fibroblast growth factors (FGFs) are controlled by trapping FGF molecules within small, closed extracellular spaces (termed microlumina), from which they have access to only a discrete collection of cells. Future studies might resolve the question whether or not collectives of CTCs harness a developmental principle to gain improved colonization.

### Loss of imprinting underlays driver roles of IGF2 in ileal neuroendocrine tumors

A recent, small intestine cancer study, provides an example of epigenetic regulation of metastasis (i.e. gene silencing). Ileal neuroendocrine tumors (I-NETs), which affect the distal portion of the small intestine, usually progress slowly, but they have often metastasized to the liver by the time the patient presents [62]. Due to robust and extensive metastasis, some patients with advanced I-NET undergo liver transplantation. The genetic driver of this disease has long remained a mystery. The overall frequency of mutations in I-NETs is rather low and I-NETs have no known mutations in oncogenes or tumor suppressor genes. Although no animal models of I-NETs have been available, the transgenic RT2 system [63], originally developed as a model of insulinomas, can model different NET subtypes if the genetic background is changed. It has recently been shown that RT2 mice can develop I-NETs on a specific genetic background. Analysis of this first I-NET animal model allowed identification of the insulin-like growth factor 2 (IGF2) as the first I-NET driver gene [64]. Elevated serum IGF2 is associated with an increased risk of developing various cancers, including colorectal. IGF2, as well as the receptor, IGF2R, is one of a relatively small number of imprinted genes in mammals. Thus, due to maternal imprinting, IGF2 is typically expressed from the paternal allele only. I-NETs generated by transgenic RT2 mice depended upon genetic background and displayed loss of imprinting (LOI) [64]. As would be expected, LOI correlated with increased *IGF2* transcription in the tumors of 57% of patients with I-NET. In summary, *IGF2* appears to be the first genetic driver of the highly metastatic but mutation-poor I-NET tumors. Importantly, LOI is expected to only double IGF2 concentrations, but this might be sufficient for tumorigenesis, which underscores the importance of growth factor homeostasis. Notably, the involvement of secreted IGF2 in metastasis has recently been reported by a study that analyzed brain pericytes producing IGF2 and promoting metastasis of breast cancer to brain [65].

### Roles for TGF-beta in the establishment of the pre-metastatic niche

The pleiotropic actions of TGF-b as a mediator of immune homeostasis and tolerance, inducer of EMT and ECM, as well as a promoter of tumor immune evasion, complicate interpretation of the influence of the TGF-b pathway on cancer progression [66]. While in early stages this pathway has tumor suppressor functions, such as cell-cycle arrest and apoptosis, in late stages TGF-b can promote tumorigenesis, including metastasis. One exemplification of the late-stage roles and the ability of TGF-b to enhance metastasis has been linked to the PMN [67]. As aforementioned, PMN formation is a stepwise process resulting from the combined systemic effects of tumor-secreted factors and exosomes. For example, according to a recent report, a tumor-exosome mediated pathway promotes liver metastasis of PDAC cells in a mechanism involving TGF-b and fibronectin [68]. The authors pre-treated naive mice with exosomes derived from normal pancreatic cells, or from PDAC cells, and later injected the PDAC cells into the portal vein of the mice to generate liver metastases. Mice pre-treated with PDAC-derived exosomes exhibited significantly more metastatic lesions in the liver, indicating that these exosomes can condition the liver towards a microenvironment able to support the growth of PDAC cells. Additional experiments revealed that uptake of PDAC-derived exosomes by Kupffer cells enhanced secretion of TGF-b and production of fibronectin by hepatic stellate cells. As a result, the newly formed fibrotic microenvironment enhanced the recruitment of bone marrow-derived macrophages. These findings suggest that tumor-derived exosomes prime the liver for metastasis.

## Perspective

- Because the overwhelming majority of cancer-associated deaths are caused by metastatic disease, understanding the complexity of this process is vital to optimizing the treatment of patients with advanced malignancies.
- Metastasis is driven by a combination of genetic drivers (mutations) and a large variety of epigenetic processes conferring a diverse collection of phenotype alterations, which offer ample opportunities for pharmacological interventions.
- Precise understanding of the metastatic process and the multiple roles played by growth factors and signaling pathways will enable effective ways to prevent, diagnose and treat metastasis.

## Abbreviations

CTCs: circulating tumor cells
DCIS: ductal carcinoma *in situ*
ECM: extracellular matrix
EMT: epithelial–mesenchymal transition
FGFs: fibroblast growth factors
HB-EGF: heparin-binding EGF-like growth factor
I-NETs: Ileal neuroendocrine tumors
LOI: loss of imprinting
LPA: lysophosphatidic acid
MET: mesenchymal-epithelial transition
NC: neural crest
NETs: neutrophil extracellular traps
PDAC: pancreatic ductal adenocarcinoma
PMN: pre-metastatic niche
SYNJ2: synaptojanin 2
TGF: transforming growth factor
